# Supplementary material for: Active site specificity profiling datasets of matrix metalloproteinases (MMPs) 1, 2, 3, 7, 8, 9, 12, 13 and 14
Source: Data Brief. 2016 Feb 22;7:299–310. doi: 10.1016/j.dib.2016.02.036 (PMC4777984; doi:10.1016/j.dib.2016.02.036)
Supplement: Supplementary file 10 — Supplementary material [file mmc10.zip › WebPICS_hMMP7_T_1%/P1prime.html]

 

PICS results


|  |  |
| --- | --- |
| **P1prime\_M**  13 in 291 sites   4.5 %    effects > 10 perc. pnts.  (vice-versa in brackets)  P2\_E: 35.2 (14.3)   P1\_S: 22.6 (12.2) |  |
